# The hidden costs of technical advancements: software and equipment troubleshooting in robotic inguinal hernia repair

**DOI:** 10.1093/jscr/rjag878

**Published:** 2026-09-29

**Authors:** Christian Martin, Christian Howells

**Affiliations:** Department of Clinical Clerkships, Ross University School of Medicine, 2300 SW 145th Avenue, Miramar, FL 33027, United States; Department of Internal Medicine, Lexington Medical Center, 2720 Sunset Boulevard, West Columbia, SC 29169, United States

**Keywords:** robotic hernia repair, technical troubleshooting, operating room efficiency, patient safety, process optimization

## Abstract

While robotic transabdominal preperitoneal (R-TAPP) hernia repairs offer excellent visibility, literature overlooks time lost to technical troubleshooting. This technical note evaluates a 30-minute pre-incision delay during a routine R-TAPP procedure, precipitated by a software synchronization error and system positioning fault. Serial system cycles, mechanical recalibrations, and hardware reboots were required for resolution. With operating room resource utilization valued between $30 and $100 per minute, this single technological latency resulted in a projected institutional deficit up to $3000 and needlessly prolonged patient anesthesia exposure. As robotic platforms become central to residency curricula, efficiency metrics must encompass technological orchestration alongside console time. We propose implementing a standardized **Robotic Pre-Incision Synchronization Protocol** for multi-disciplinary staff and urge manufacturers to design accelerated diagnostic interfaces to safeguard hospital financial reserves and optimize patient safety.

## Introduction

Robotic-assisted surgical platforms have significantly enhanced the management of inguinal hernias, offering clinicians three-dimensional stereoscopic visualization, tremor filtration, and multi-jointed wrist articulation. Despite these clear technical advantages, comparative trials between laparoscopic and robotic approaches heavily prioritize post-operative clinical endpoints, often excluding the critical time vectors lost to hardware initialization and software troubleshooting [1].

While intraoperative technological latency is frequently minimized as a minor logistics challenge, its cumulative impact across a high-volume surgical service presents substantial financial burdens and latent clinical risks [2]. This technical note evaluates a discrete operational delay caused by system synchronization failures during a robotic transabdominal preperitoneal (R-TAPP) hernia repair and outlines an actionable institutional troubleshooting protocol to mitigate iatrogenic workflow interruptions.

## Case presentation / technical note

During a scheduled R-TAPP inguinal hernia repair at an academic teaching hospital, a major operational interruption occurred prior to the first surgical incision. Following patient positioning, induction of general anesthesia, and initial equipment docking, the robotic console and patient cart threw a critical system configuration error. This error was characterized by a software synchronization failure between the master console controllers and a hardware system positioning fault on the primary patient cart arm.

The resolution of this technological glitch required a 30-minute delay, spent entirely on systematic platform reboots, mechanical recalibration of the optical and arm sensors, and consecutive software diagnostic cycles. During this window, the surgical team, nursing staff, and anesthesia providers remained completely stationary, waiting for system initialization.

In standard laparoscopic interventions, instrumentation achieves functional readiness within minutes, carrying a near-zero incidence of catastrophic software orchestration failure. Peer-reviewed economic modeling estimates modern operating room utilization costs between $30 and $100 per minute [2]. Consequently, a 30-minute technological latency translates directly to an institutional deficit of ~$900 to $3000 per surgical event.

Furthermore, this delay extended the patient’s exposure to general anesthesia and muscle relaxants without adding any clinical value. Across a high-volume municipal or academic hospital system executing multiple robotic cases per day, unmitigated software and positioning latencies compound into a significant financial strain and an unacceptable patient safety liability [1].

## Discussion

To address the hidden structural expenses of advanced surgical platforms, institutional performance metrics must evolve to track total room utilization time rather than console time alone. As robotic platforms become a core competency in general surgery training, hospitals must move away from ad-hoc troubleshooting and adopt proactive workflow interventions [3, 4].

We propose the implementation of a standardized, multi-disciplinary **Robotic Pre-Incision Synchronization Protocol (RPSP)**. This protocol establishes a strict, time-delimited checklist executed by the circulating nurse, surgical technologist, and console surgeon prior to patient draping:

**Table:** 

Protocol step	Responsible party	Action items	Target window
1. Orchestration Verification	Circulating Nurse	Verify central software patch alignment; confirm active link between console and patient cart.	Pre-Induction
2. Mechanical Clearing	Surgical Technologist	Validate arm articulation clearances; clear optical sensor paths of debris or shroud interference.	Post-Positioning
3. Diagnostic Cycle	Console Surgeon	Execute rapid-cycle platform diagnostics; confirm tool-tracking alignment prior to docking.	Pre-Incision

Implementing this structured layout ensures that minor software latencies are identified and mitigated before the patient is under anesthesia. Additionally, a clear responsibility falls on medical device manufacturers to optimize system architecture, developing faster diagnostic interfaces and rapid-reboot recovery modes to minimize the duration of system cycles.

## Conclusions

As modern general surgery transitions past conventional laparoscopy, the time lost to hardware and software maintenance can no longer be ignored as an unmeasurable variable. Implementing standardized, rapid-cycle troubleshooting protocols like the RPSP protects hospital financial resources and shields patients from prolonged, unnecessary anesthetic exposure due to technical platform failure.

## References

[1] Köckerling F, Schug-Pass C. Economic analysis of the robotic approach to inguinal hernia repair (r-TAPP) vs laparoscopic approach (l-TAPP). Surg Endosc 2024;38:2318–25.

[2] Childers CP, Maggard-Gibbons M. Understanding costs of care in the operating room. JAMA Surg 2018;153:e176233. 10.1001/jamasurg.2017.6233 PMC5875376 29490366

[3] Zens JJ . Initial trajectory and outcomes of a robotics program for inguinal hernia repair. J Robot Surg 2025;19:117–22.

[4] Morrell DJ, Tholey RM, Juza RM et al. Robotic inguinal hernia repair outcomes: operative time, cost, and learning curve. Surg Endosc 2020;34:4320–8.

